# Super-Resolution Imaging of ESCRT-Proteins at HIV-1 Assembly Sites

**DOI:** 10.1371/journal.ppat.1004677

**Published:** 2015-02-24

**Authors:** Jens Prescher, Viola Baumgärtel, Sergey Ivanchenko, Adriano A. Torrano, Christoph Bräuchle, Barbara Müller, Don C. Lamb

**Affiliations:** 1 Physical Chemistry, Department of Chemistry, Nanosystem Initiative Munich (NIM), Center for Integrated Protein Science Munich (CiPSM) and Center for NanoScience (CeNS), Ludwig-Maximilians-Universität München, Munich, Germany; 2 Department of Infectious Diseases, Virology, Universitätsklinikum Heidelberg, Heidelberg, Germany; Vanderbilt University School of Medicine, UNITED STATES

## Abstract

The cellular endosomal sorting complex required for transport (ESCRT) machinery is involved in membrane budding processes, such as multivesicular biogenesis and cytokinesis. In HIV-infected cells, HIV-1 hijacks the ESCRT machinery to drive HIV release. Early in the HIV-1 assembly process, the ESCRT-I protein Tsg101 and the ESCRT-related protein ALIX are recruited to the assembly site. Further downstream, components such as the ESCRT-III proteins CHMP4 and CHMP2 form transient membrane associated lattices, which are involved in virus-host membrane fission. Although various geometries of ESCRT-III assemblies could be observed, the actual membrane constriction and fission mechanism is not fully understood. Fission might be driven from inside the HIV-1 budding neck by narrowing the membranes from the outside by larger lattices surrounding the neck, or from within the bud. Here, we use super-resolution fluorescence microscopy to elucidate the size and structure of the ESCRT components Tsg101, ALIX, CHMP4B and CHMP2A during HIV-1 budding below the diffraction limit. To avoid the deleterious effects of using fusion proteins attached to ESCRT components, we performed measurements on the endogenous protein or, in the case of CHMP4B, constructs modified with the small HA tag. Due to the transient nature of the ESCRT interactions, the fraction of HIV-1 assembly sites with colocalizing ESCRT complexes was low (1.5%-3.4%). All colocalizing ESCRT clusters exhibited closed, circular structures with an average size (full-width at half-maximum) between 45 and 60 nm or a diameter (determined using a Ripley’s L-function analysis) of roughly 60 to 100 nm. The size distributions for colocalizing clusters were narrower than for non-colocalizing clusters, and significantly smaller than the HIV-1 bud. Hence, our results support a membrane scission process driven by ESCRT protein assemblies inside a confined structure, such as the bud neck, rather than by large lattices around the neck or in the bud lumen. In the case of ALIX, a cloud of individual molecules surrounding the central clusters was often observed, which we attribute to ALIX molecules incorporated into the nascent HIV-1 Gag shell. Experiments performed using YFP-tagged Tsg101 led to an over 10-fold increase in ESCRT structures colocalizing with HIV-1 budding sites indicating an influence of the fusion protein tag on the function of the ESCRT protein.

## Author Summary

Viruses hijack the cellular machinery to complete their life cycle. In the case of HIV-1, the endosomal sorting complex required for transport (ESCRT) is recruited by nascent viruses to release themselves from infected cells. Currently, there has been an intense amount of research on how the ESCRT machinery induces viral release. Using super-resolution imaging with endogenous ESCRT proteins or ESCRT proteins containing a small tag, we are able to provide insight into how ESCRT leads to budding of HIV-1. Super resolution imaging of the early ESCRT factors Tsg101 and ALIX, as well as later factors CHMP4B and CHMP2A, also showed condensed, circular structures with diameters of roughly 60 to 90 nm. The cluster sizes were significantly smaller than that of the HIV-1 bud and the distribution of cluster sizes that colocalized with nascent HIV-1 assembly sites were narrower than for non-colocalizing structures. This indicates that the point of interaction between the ESCRT machinery and the HIV-1 assembly site is in the bud neck.

## Introduction

The budding of HIV-1 at the plasma membrane of a virus-producing cell relies on recruitment of, and interaction with, various host cell factors. Initial viral bud formation is primarily induced by assembly of plasma membrane associated Gag molecules into a hexagonal lattice that attains an outward curvature through the introduction of irregular lattice defects (reviewed in [1–3]). However, for the final membrane remodeling steps leading to fission, HIV-1 relies on the cellular endosomal sorting complex required for transport (ESCRT) (reviewed in [4–6]) that is mechanistically involved in various cellular membrane bending and separation processes, including the formation of multivesicular bodies (MVB) or cytokinesis (reviewed in [7–9]). ESCRT consists of four different sub-complexes (ESCRT-0 to ESCRT-III) and associated factors such as VPS4 and the ALG-2 interacting protein X (ALIX) (reviewed in [9,10]), HIV-1 employs ESCRT-I, as well as components of ESCRT-III and the AAA ATPase Vps4 in the viral budding process [11–16]. Alternatively to ESCRT-I, ALIX may also serve to recruit ESCRT-III to HIV-1 budding sites [11,17,18].

**Fig 1:**
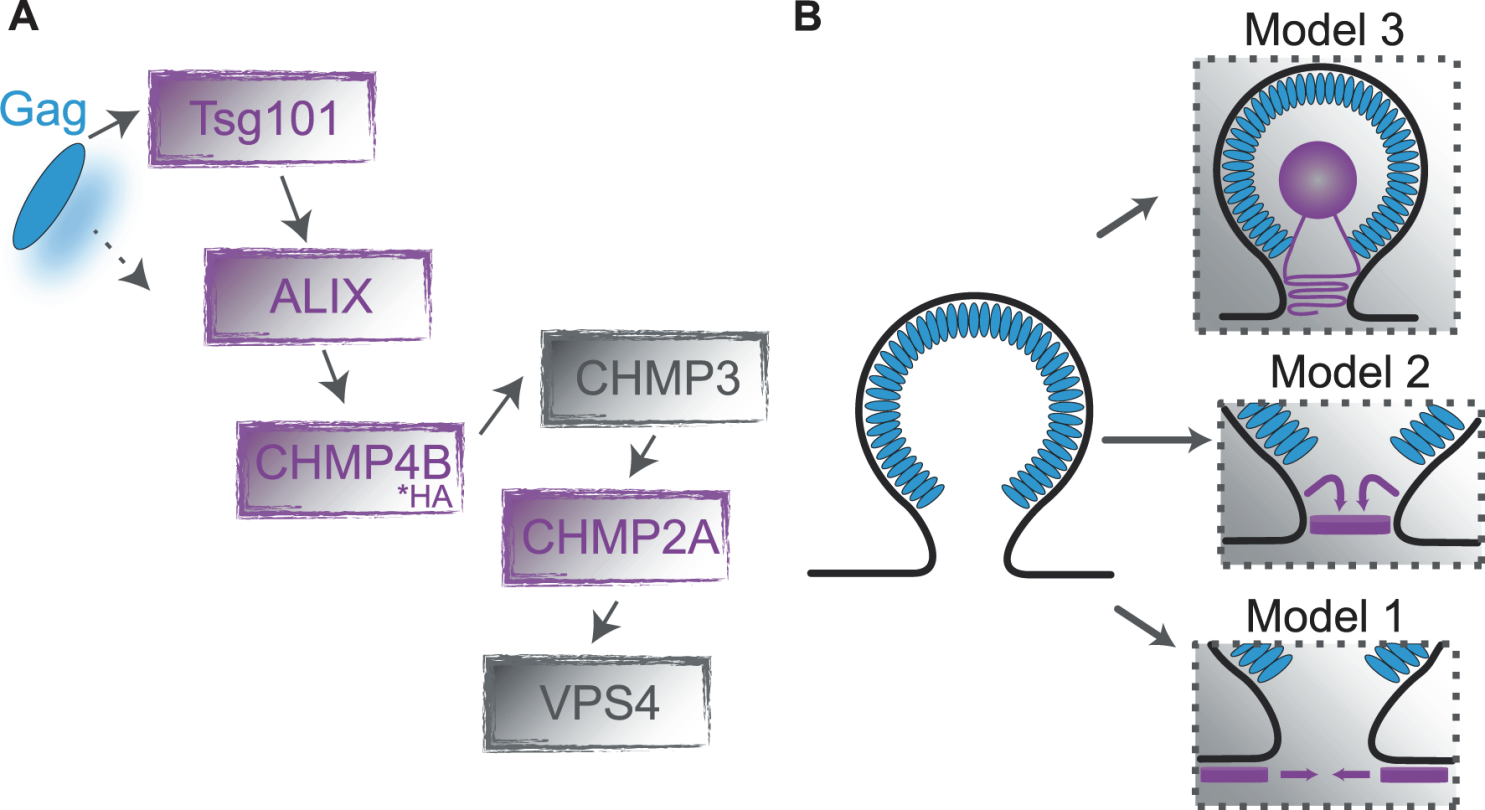
Gag-ESCRT Interactions. (**A**) A simplified schematic of the interaction scheme between HIV-1 assembly sites and the ESCRT recruitment pathway. The constructs analyzed in this study are highlighted in purple. (**B**) An overview of the proposed models of membrane scission where ESCRT factors interact either via external constriction from the cytosol (cytosolic model, Model 1), via internal constrict of the budding virus in the neck region (neck model, Model 2) or from within the bud (bud model, Model 3).

The ESCRT machinery is recruited via the C-terminal p6-domain of HIV-1 Gag. This small protein comprises two so-called late domain (L-domain) motifs that bind to the central ESCRT-I component tumor susceptibility gene 101 (Tsg101) [16,19–21] or to ALIX [11,17,22], respectively. Both ESCRT-I and ALIX can serve to recruit ESCRT-III to the viral budding site. The Tsg101 interacting PT/SAP motif has been identified as the major determinant of HIV-1 release; its mutation affects particle release and leads to the arrest of late budding structures at the plasma membrane [16,23,24]. In contrast, the ALIX-interacting LYPXL motif apparently serves a partly redundant, auxiliary function in the case of HIV-1 [18]. Although mutation of the ALIX binding site has less pronounced effects on HIV-1 release compared to mutation of PT/SAP in model cell lines [25,26], overexpression of ALIX fragments can exert a dominant-negative effect on virus budding [17,27]. Furthermore, the release of HIV-1 mutants unable to interact with Tsg101 can be rescued by overexpression of ALIX [25,28]. Thus, ESCRT-III recruitment in most cell systems studied is predominantly mediated by ESCRT-I via an ESCRT-II independent pathway, but an alternative route involving direct interaction of ALIX with the ESCRT-III component CHMP4 [17,25,29] is possible (Fig. 1A). Besides their function in recruiting ESCRT-III, both ESCRT-I and ALIX may play a role in membrane bending or remodeling [30].

The formation and re-organization of membrane bound ESCRT-III multimers is presumed to be the central driving force for membrane remodeling during the fission process. In the past years, various *in vivo* and *in vitro* studies of cytokinesis [8,31], as well as HIV-1 release [5,6,32] and multivesicular budding [7], have led to different models for the mechanism of membrane constriction and fission (Fig. 1B). One of the early proposed models was from Hanson *et al*. who observed circular and spiral-shaped structures of endogenous ESCRT-III protein membrane assemblies on the cytoplasmic side of the plasma membrane using deep-etch electron microscopy [33]. This and other studies [34,35] suggested models in which spiraling or constricting ESCRT-III filaments slide past each other resulting in membrane constriction (Fig. 1B, model 1). A second family of models presume that ESCRT-III filaments assemble within the budding neck [7] and, together with other ESCRT components, form dome- [36,37], whorl- [38], circular or spiral-shaped structures [31,34,39–43]. In these models, constriction of the plasma membrane occurs from within the neck (Fig. 1B, Model 2). Remodeling of multimeric CHMP4 complexes could eventually lead to narrowing of the bud neck from within the fission neck and spontaneously induce membrane fission when the neck diameter reaches a lower threshold [36,44]; alternatively, the ESCRT associated ATPase VPS4 has been proposed to be actively involved in membrane remodeling [13,45]. Two other ESCRT-III proteins, CHMP2 and CHMP3, both of which stabilize the neck from within by forming a dome-structure, may also play a supportive role [36,37,39,42]. Electron micrographs of nascent HIV-1 particles arrested at a late stage of budding by depletion of CHMP2 showed ring-like striations situated within the bud neck[14], favoring an internal constriction model. Further support for models of type 2 is provided by *in vitro* experiments using giant unilamellar vesicles (GUVs) and purified ESCRT-III proteins. These studies indicated a direct involvement of other ESCRT-III components in the initial membrane neck formation during HIV-1 assembly [34,46]. Studies from Lata *et al*. [37] showed the formation of large tubular structures by CHMP2-CHMP3 heteromeric complexes suggesting that CHMP2-CHMP3 clusters form a dome-like assembly inside HIV-1 budding neck, which is supported by computational thermodynamic calculations [36]. Recently, Hanson and coworkers used deep-etch electron microscopy to analyze membrane associated ESCRT-III assemblies arrested by VPS4 depletion, reporting spiral shaped arrangements with an average diameter of ~110 nm [40]. Nascent HIV-1 buds in Vps4 depleted cells were found to be encircled by ESCRT-III filaments. These findings suggested that Vps4 may be required for the transition of a ring-like structure encircling the bud neck to a growing spiral that constricts the bud neck from within. Finally, van Engelenburg *et al*. [47] recently proposed a new model predicting membrane constriction to be mediated from within the virus bud (Fig. 1B, Model 3). Using two-color 3D-iPALM super-resolution microscopy on eGFP protein fusions of Tsg101 and the ESCRT-III proteins CHMP2A and CHMP4B, they found that all factors localized in the center of a nascent Gag bud. This result contrasted all previously described models that suggested ESCRT-III proteins driving membrane fission from around or below the nascent virus-like particles (VLPs), as well as biochemical analyses, which did not detect ESCRT-III proteins in purified HIV-1 particles. It thus remains to be clarified, whether the surprising findings might be related to altered localization or multimer arrangements of the GFP-tagged ESCRT components.

To resolve this issue and investigate the mechanism of membrane scission under more physiological conditions, we measured the size and localization of endogenous ESCRT protein assemblies at assemblies of HIV-1 Gag expressed in the complete viral context [48]. To avoid potential alterations in ESCRT localization and binding caused by fluorescent protein (FP) tags, we used antibodies for immunodetection of endogenous ESCRT proteins or employed a small epitope tag where suitable antibodies were not available. Super-resolution fluorescence microscopy was employed to analyze complexes smaller than the diffraction limit of optical microscopy. This approach allowed us to monitor hundreds of μm^2^ in a single image and thereby obtain statistically relevant information on the comparatively low number of interactions observed with endogenous ESCRT proteins in the absence of an experimentally induced budding arrest. We investigated the early-acting factors Tsg101 and ALIX as well as the late-acting ESCRT factors CHMP4B and CHMP2A known to assemble into high-molecular structures at the membrane (Fig. 1A). Super-resolution images of each of these ESCRT and ESCRT-related protein assemblies colocalizing with single HIV-1 budding sites showed a condensed circular spot with a full-width at half-maximum (FWHM) between ~ 45 nm – 60 nm or a diameter (determined using a Ripley L-function analysis [49]) of approximately ~ 60 – 100 nm. Our results are consistent with an internal membrane constriction mechanism driven by protein assemblies smaller than HIV-1 budding structures and more similar to the size of the budding neck. In addition, when performing experiments in cells overexpressing fluorescent protein fusions of the ESCRT protein Tsg101, an increase of over 10-fold in the colocalization of ESCRT complexes with HIV-1 assembly sites was observed indicating that the function and potentially also the structure of the ESCRT complexes are altered by the large fusion protein label.

## Results and Discussion

### Labeling budding sites for super-resolution microscopy

To put the super-resolution images of the various ESCRT factors in the context of the HIV assembly sites, we first measured the size of assembly sites at the plasma membrane with Photoactivation Localization Microscopy (PALM) in TIRF mode using mEos-labeled Gag expressed in the viral context (See Supporting Information). The average size of the assembly sites was found to be 116 ± 36 nm full-width at half-maximum (FWHM) or a diameter of 141 ± 41 nm from the Ripley-L analysis (See Supporting Results and S1 and S2 Figs. for details). This is similar to the size of released HIV-1 particles determined by cryo-electron microscopy [50,51] and previous super-resolution studies [52–54]. To verify that fixation and permeabilization of the HeLa cells does not lead to artifacts, we also performed live-cell PALM experiments with the mEos-labeled Gag constructs expressed in the viral context and obtained similar results (a FWHM of 108 ± 35 nm or a diameter of 152 ± 37 nm from the Ripley-L analysis, S2 Fig.). As the relationship between results of the Ripley analysis and actual cluster size is very sensitive to labeling and cluster densities [55], we chose to give the cluster sizes as the FWHM of our super-resolution analysis, which is slightly smaller than the actual diameter.

The accuracy of measured super-resolution images depends on the labeling efficiency and effectivity of the antibody staining. To determine the efficiency of labeling protein structures within the bud, we performed experiments with an eGFP tagged version of the viral accessory protein Vpr. Vpr is recruited to the HIV-1 assembly sites by interacting with the C-terminal p6 domain of the polyprotein precursor Pr55^Gag^ inside the bud [56–58]. eGFP.Vpr was co-expressed with HIV-1 Gag alone to avoid expression of endogenous Vpr; for this, we made use of a previously described Rev-independent Gag expression construct [59], carrying an FP tag at the C-terminus. HeLa cells were transiently co-transfected with an equimolar ratio of plasmids encoding HIV-1 Gag and a mCherry-tagged derivative, respectively, together with peGFP.Vpr and fixed at 18–20 hpt. For super-resolution imaging, eGFP.Vpr was marked using a primary polyclonal anti-GFP antibody and fluorescently-labeled secondary-antibody. TIRF images of cells expressing both pEGFP.Vpr (S3A Fig., left panel), Gag:Gag.mCherry (1:1) (S3A Fig., middle panel) and anti-GFP immunostaining (S3A Fig, right panel) revealed a high number of distinct eGFP.Vpr protein assemblies strongly colocalizing with HIV^mCherry^ budding sites (S3B–S3C Fig.) as would be expected for functional recruitment of Vpr during Gag assembly. The primary/secondary antibody complexes labeled the majority of HIV-1 assembly sites displaying a detectable eGFP.Vpr signal, confirming that a significant fraction of proteins present within the nascent bud is accessible to immunostaining. Fitting a Gaussian function to the cross-section profile of 49 colocalizing spot-like super-resolution reconstructions of immunodetected Vpr protein assemblies yielded an average FWHM of 56 ± 12 nm (S3D Fig.), smaller than the VLP diameter determined by PALM imaging of Gag.mEos, consistent with a central localization of eGFP.Vpr within the bud. A Ripley analysis of our super resolution data revealed similar results to the experiments performed by Lehman *et al* [53] with a diameter of 95 ± 53 nm.

### Imaging and size characterization of Tsg101 clustering at HIV-1 assembly sites using Stochastical Optical Reconstruction Microscopy (STORM)

**Fig 2:**
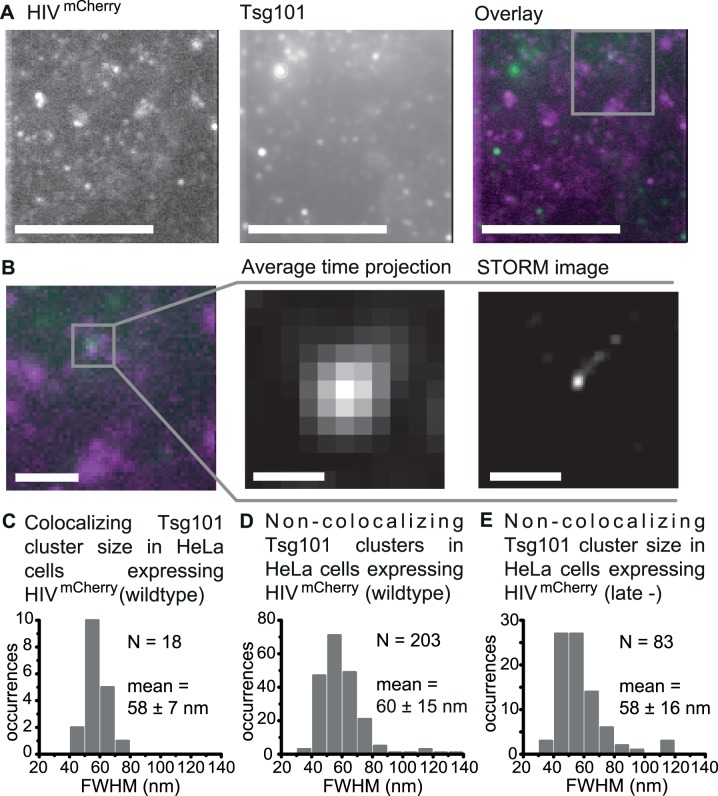
Super-resolution images of Tsg101 at HIV-1 assembly sites. HeLa cells were transfected with HIV:HIV^mCherry^ and endogenous Tsg101 was immunostained at 14 – 15 h post transfection. (**A**) Cells were imaged using TIRFM. A time projection of the TIRFM images are shown (HIV^mCherry^, *left panel*; Tsg101, *middle panel*; Overlay, magenta: HIV^mCherry^, green: Tsg101, *right panel*), scale bars: 10 μm. (**B**) *Left panel*. A zoomed image of the region highlighted in panel **A**. Scale bar: 2 μm. *Middle panel*. A zoomed image of the individual colocalizing Tsg101 cluster highlighted in the left panel. Scale bar: 500 nm. *Right panel*. A drift-corrected STORM image of the Tsg101 complex shown in the middle panel. Scale bar: 500 nm. (**C**) The size distribution of all Tsg101 structures colocalizing with HIV^mCherry^, revealing an average cluster size of 58 ± 7 nm. (**D**) Size distribution of all non-colocalizing Tsg101 clusters in cells expressing HIV^mCherry^ revealing an average cluster size of 60 ± 15 nm or (**E**) or in cell expressing HIV^mCherry^ late- revealing average cluster size of 58 ± 16 nm. Number *N* represents the number events contributing to the respective histogram.

Having determined the diameter of HIV-1 budding sites in the context of our experiment, we characterized the size and structure of membrane associated endogenous Tsg101 protein assemblies acting as recruitment factors during the early stages of HIV-1 assembly (Fig. 1A) using Stochastical Optical Reconstruction Microscopy (STORM). HeLa cells were transiently transfected with the previously characterized full viral construct for HIV^mCherry^ [48] at a ratio of 1:1 with unlabeled HIV construct to mark the position of HIV-1 budding sites. Endogenous Tsg101 proteins were directly visualized 14–15 h post transfection by adding primary monoclonal anti-Tsg101 antibodies and appropriate secondary antibodies that were labeled with the activator-reporter dye pair Alexa Fluor 488-Cy5. TIRF microscopy images of fixed cells revealed distinct HIV-1 budding sites and Tsg101 protein assemblies at the membrane (Fig. 2A-2B) that were classified into colocalizing and non-colocalizing structures. Large fluorescent Gag assemblies whose appearance did not correspond to individual budding sites were excluded from the analysis. In 14 cells analyzed, 18 Tsg101 clusters were found to colocalize with HIV-1 assembly sites, corresponding to a colocalization percentage of 1.8% with respect to all detected assembly sites. To verify that the low number of colocalizations is not due to inefficient labeling of the epitope, we performed experiments with YFP-tagged Tsg101 (discussed in detail below). Over 60% of the Tsg101 complexes observable via the YFP signal were also detected using an anti-Tsg101/secondary antibody construct. Hence, the low number of colocalizations can be attributed to the transient, dynamic nature of the HIV-1 budding process and ESCRT recruitment [13,60]. Reconstructions based on 2D Gaussian localization revealed that all colocalizing Tsg101 structures appeared as small, condensed, circular spots (Fig. 2B). The size (FWHM) of the 18 colocalizing spot-like Tsg101 clusters was between 40 and 75 nm (Fig. 2C) with a mean value of 58 ± 7 nm. This is significantly smaller than the previously determined mean HIV-1 bud size of 116 ± 36 nm (Reference).

Untransfected HeLa cells stained with the same set of antibodies showed condensed, roundish Tsg101 protein assemblies at the cell membrane (S4A Fig.) resembling those observed in HIV-1 transfected cells (Fig. 2). This is in agreement with Welsch *et al*. who used quantitative immuno-EM to show that ~15% of all Tsg101 proteins were present at the plasma membrane of untransfected immune cells and that the distribution of Tsg101 between plasma and intracellular membranes did not significantly change upon HIV-1 infection [61]. Our findings confirm the capability of Tsg101 to assemble at the plasma membrane due to processes unrelated to HIV-1 budding. It also highlights the necessity to verify the colocalization of ESCRT structures with HIV assembly sites and thus to exclude structures in the analysis caused by other cellular events. We also performed experiments with an HIV^mCherry^ late- variant of HIV^mCherry^ comprising a mutation of the PT/SAP motif. Consistent with what is expected, we did not observe a single structure that colocalized with the more than 210 HIV-1 assembly sites detected.

If the neck of the budding structure was limiting the size of the Tsg101 containing protein assemblies at budding sites, one might expect a more narrow size distribution than for non-colocalizing structures. Indeed, whereas the average diameter of non-colocalizing clusters was similar (60 ± 15 nm), the STORM analysis revealed a broader size distribution than for the colocalizing Tsg101 clusters, ranging from 35 to 135 nm (Fig. 2D). Likewise, a broader size distribution was also observed for 83 non-colocalizing Tsg101 clusters detected at the membrane of recruitment defective HIV^mCherry^ (late-) expressing cells (Fig. 2E and S4C Fig.). This observation suggests that the dimensions of the Tsg101 complexes specifically recruited to HIV-1 budding sites may be confined.

### Distribution and clustering of membrane-bound ALIX during HIV-1 budding

**Fig 3:**
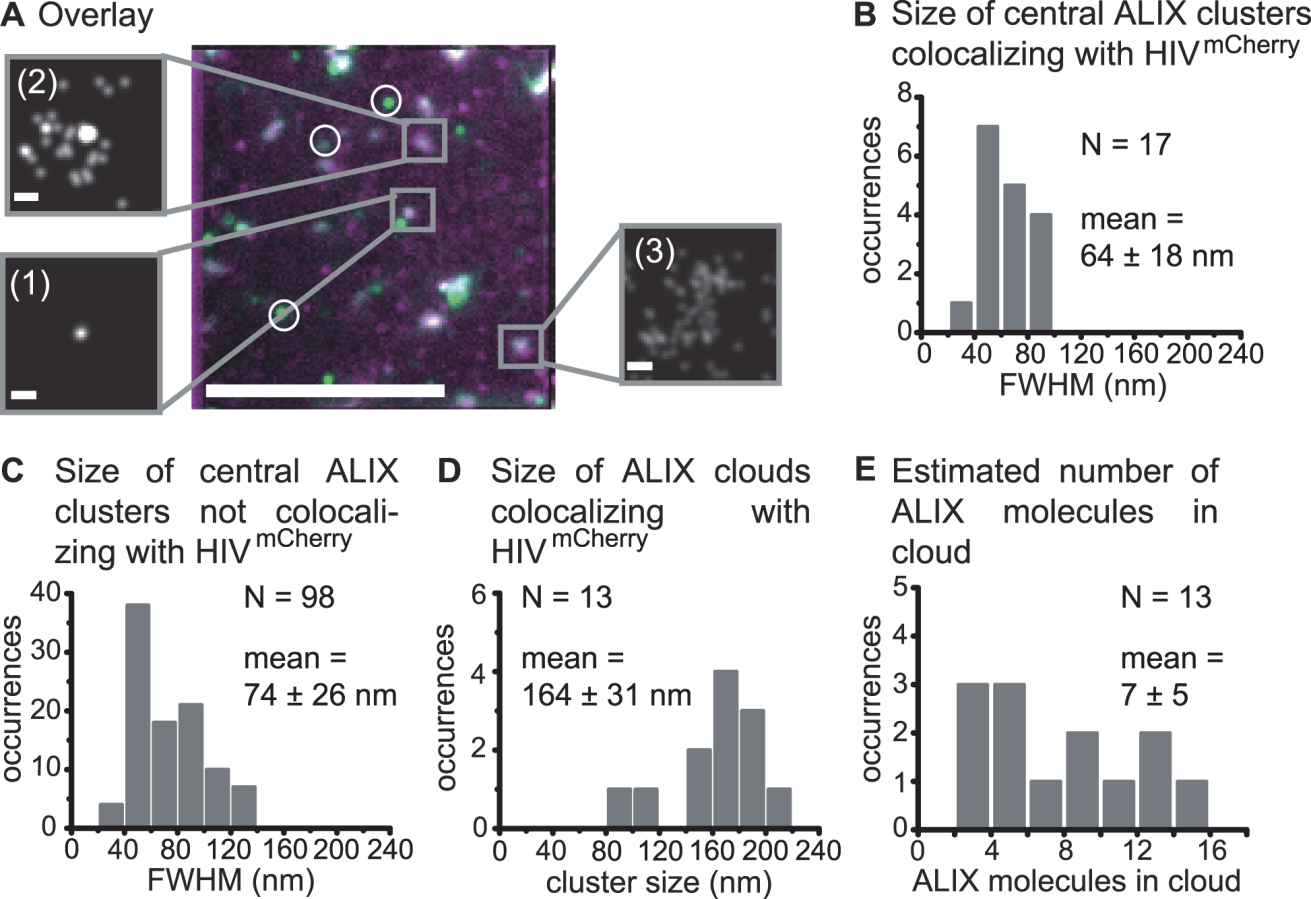
Super-resolution imaging of endogenous ALIX at HIV-1 assembly sites. HeLa cells were transfected with HIV:HIV^mCherry^ and endogenous ALIX was immunostained at 14 – 15 h post transfection. Cells were imaged by TIRFM. (**A**) A time projected image of ALIX (green) overlaid with TIRF image of HIV^mCherry^ assembly sites (magenta) is shown. Circles indicate non-colocalizing ALIX clusters and the rectangles highlight different colocalized ALIX classes. The crops show drift-corrected super-resolution STORM images of the three identified object classes: condensed ALIX structures without a surrounding cloud (1), ALIX structures with a central, condensed spot surrounded by a cloud-like structure (2) and diffuse ALIX membrane assemblies without a central spot (3). Scale bars: 10 μm (large image), 100 nm (crops). (**B**) Size distribution of all central spots of colocalizing ALIX protein clusters with an average cluster size (FWHM) of 64 ± 18 nm. (**C**) Size distribution of all central spots of non-colocalizing ALIX protein clusters with an average cluster size (FWHM) of 74 ± 26 nm. (**D**) Cloud cluster size characterization for all cloud structures of ALIX colocalizing with HIV^mCherry^ obtained by Ripley’s cluster analysis with an average diameter of 164 ± 31 nm. (**E**) The distribution of the minimum number of ALIX proteins detected per cloud estimated from the super-resolution data.

We also investigated the ESCRT-related protein ALIX, another early-acting factor in HIV-1 budding (Fig. 1A), because of its key function in the ESCRT-recruitment process and its potential role in stabilizing the CHMP4-scaffold. HeLa cells were transiently transfected with pCHIV:pCHIV^mCherry^ and endogenous ALIX was directly stained 14–15 h post transfection using primary anti-ALIX antibodies labeled with the activator-reporter dye pair Alexa Fluor 488-Cy5. TIRF images of the fixed samples revealed several individual HIV-1 budding sites at the cell membrane as well as immunostained ALIX protein assemblies, which were manually classified into colocalizing and non-colocalizing clusters (Fig. 3A). Three distinct classes of colocalizing ALIX structures were identified from super-resolution data, namely condensed spots (Fig. 3A, crop (1)), condensed spots with a surrounding, diffuse cloud-like structure (Fig. 3A, crop (2)) and diffuse cloud-like structures without a central spot (Fig. 3A, crop (3)For the non-colocalizing clusters, only condensed spots were observed. Super-resolution STORM reconstructions of membrane associated ALIX structures in untransfected cells exclusively exhibited condensed spots of ALIX (S5A Fig.), verifying that the cloud-like ALIX structures were budding site specific. Thus, the non-colocalizing round ALIX structures in cells expressing HIV^mCherry^ (Fig. 3A, circles) likely represent ALIX localized with the plasma membrane in the course of other cellular processes, which is again supported by quantitative EM analyses showing ALIX localized with the plasma membrane of uninfected cells [61]. ALIX is known to associate with so-called exosomes [4,62], intraluminal vesicles of multivesicular bodies (MVBs), that fuse with the plasma membrane to release their content for cell-cell communication [63]. Thus, the spot-like assemblies of ALIX in cells not expressing HIV or non-colocalizing with HIV-1 budding sites might potentially be attributed to exosome-related ALIX structures captured during the process of membrane secretion.

In the 10 cells analyzed, a total of 18 ALIX clusters with or without cloud structures were found to colocalize with HIV^mCherry^. In one case, it was not possible to clearly separate the central spot from the cloud structure; this site was excluded from further analysis. With the remaining 17 ALIX colocalizing structures, 3.4% of the HIV-1 assembly sites had an associated ALIX cluster, which is similar to the colocalization percentage observed for Tsg101. The FWHM of the condensed central ALIX spot ranged from 34 to 94 nm (Fig. 3B, S5B Fig.) with an average value of 64 ± 18 nm. Again, this is significantly smaller than the mean size of the HIV-1 bud. Both early-acting factors Tsg101 and ALIX appeared to form compact protein assemblies inside the confining structure of the budding neck. The localization of many ALIX proteins inside the budding neck would be necessary for any kind of neck stabilizing mechanism during HIV-1 budding or membrane scission. This is in good agreement with the proposed stabilizing function of ALIX in addition to its involvement in recruitment of downstream ESCRT factors. Analysis of the non-colocalizing ALIX clusters showed a broader size distribution ranging from 30 nm up to more than 130 nm (Fig. 3C)

Thirteen out of the 17 (76%) detected ALIX membrane clusters colocalizing with HIV-1 budding sites (S1 Table) displayed an additional diffuse cloud-like distribution of ALIX proteins surrounding the central condensed spot. This suggests that ALIX molecules, which directly interact with the LYPX_n_L or LX_n_LF L-domains of the NC domain of Gag [17,64], are taken up into the HIV-1 bud. Incorporation of ALIX molecules in released virus like particles has already been shown using biochemical analyses [11,65–68]. Any unspecific membrane-association of ALIX molecules can be excluded due to the presence of the auto-inhibitory domain in ALIX [69–71]. When ALIX molecules are taken up into the bud, the distribution of ALIX molecules should be consistent with the size of a nascent HI virion. Since the number of ALIX specific signals detected in the area of the clouds was too low to apply a 2D-Gaussian fit, we performed a Ripley’s L-function analysis [49]. This analysis yielded cluster sizes of 95 to 210 nm with a mean value of 164 ±31 nm for the colocalizing ALIX cloud-like structures (Fig. 3D, S5C Fig.). This is comparable to the distribution of HIV-1 bud sizes ranging from 80 to 180 nm with an average value of 141 ± 41 nm, when applying the same Ripley-L analysis. In addition, the size of the cloud structure shows a relatively narrow size distribution with no strong outliers.

From our data, we also made a rough estimation of the minimum number of ALIX molecules incorporated into the virus Gag shell. Only the molecules were counted that were distinct from the central cluster and fell within the size of the ALIX cloud estimated by the Ripley’s L-function. As the same fluorophore can be photoactivated several times, reoccurring molecules that localized within our resolution of 40 nm of each other were counted as a single molecule. The distribution of ALIX molecules ranged from 2 up to 15 per cloud (Fig. 3E) with a mean value of 7 ± 5 molecules. Since multiple ALIX molecules may exist within a distance of 40 nm, this represents a minimum estimation of the number of ALIX molecules within the bud.

### Super-resolution imaging of CHMP4B lattices colocalizing with HIV-1 assembly sites

In view of the different models for membrane fission, it was of particular interest to investigate the downstream ESCRT factors directly involved in the membrane scission process. CHMP4B, in contrast to the CHMP4A or CHMP4C isoforms, has a direct impact on viral infectivity and release as established from siRNA knockdown experiments [14]. Based on structural data, CHMP4B can be directly recruited to the HIV-1 assembly site via the Bro-domain of the early-acting factor ALIX (Fig. 1A) [29]. Therefore, we extend our studies on the membrane scission machinery to CHMP4B. As no antibodies against CHMP4B suitable for super-resolution imaging were available, we employed a version of the human isoform CHMP4B tagged with the small hemagglutinin (HA) tag that was subsequently immunostained with primary monoclonal anti-HA antibodies labeled with Alexa Fluor 488 and Cy5. With use of the HA tag, we minimize the dominant negative effects on ESCRT recruitment that were reported for CHMP4 isoforms fused to fluorescent proteins [11,33].

**Fig 4:**
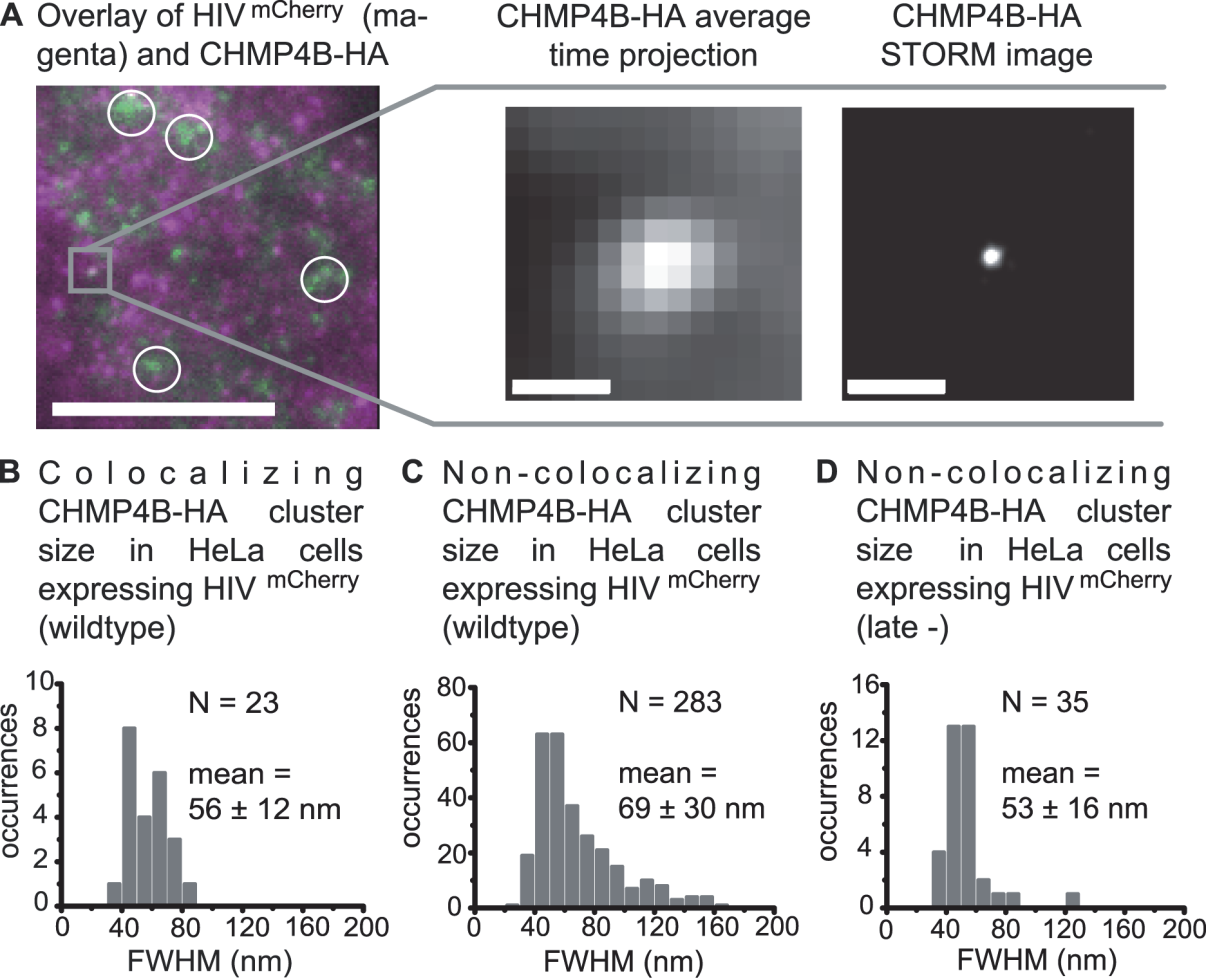
Super-resolution imaging of CHMP4B-HA clusters. HeLa cells were transfected with both HIV:HIV^mCherry^ and CHMP4B-HA and CHMP4B-HA was detected by immunostaining 14–15 h post transfection. Cells were imaged by TIRFM. (**A**) *Left panel*. A time projected overlay of HeLa cells transfected with HIV^mCherry^ (magenta) and CHMP4B-HA (green). The circles indicate partially diffuse CHMP4B clusters at the membrane, which are ignored in further analyses. Scale bar: 10 μm. *Middle panel*. A zoomed-in image of a selected CHMP4B-HA cluster (highlighted in grey in the left panel) colocalizing with HIV^mCherry^. *Right panel*. The corresponding drift-corrected STORM image. Scale bars: 500 nm (**B**) Size distribution of all CHMP4B-HA structures colocalizing with HIV^mCherry^ with an average cluster size (FWHM) of 56 ± 12 nm. (**C**) Size distribution of all non-colocalizing CHMP4B-HA clusters in cells co-expressing HIV^mCherry^ wildtype with an average cluster size (FWHM) of 69 ± 30 nm. (**D**) Size distribution of all non-colocalizing CHMP4B clusters in cells co-expressing HIV^mCherry^ late- with an average cluster size (FWHM) of 53 ± 16 nm.

In HeLa cells expressing CHMP4B-HA and HIV:HIV^mCherry^, both colocalizing and non-colocalizing CHMP4B protein clusters were observed around 14–15 h post transfection (Fig. 4A). Considering the relatively high density of detected CHMP4 protein clusters on the plasma membrane (Fig. 4A), the overall colocalization percentage of 1.5% for CHMP4B with respect to HIV-1 assembly sites is low. The low proportion of colocalizations detected in steady state is consistent with the transient recruitment of CHMP4B to the membrane for a period of approximately 2–3 min during the complete HIV-1 budding process, as shown by Jouvenet *et al*. [13] in live-cell imaging studies.

Analysis of the 23 CHMP4B-HA clusters colocalizing with HIV-1 assembly sites showed a narrow cluster size (FWHM) distribution ranging from 35 to 85 nm with a mean value of 56 ± 12 nm (Fig. 4B). Analysis of the non-colocalizing CHMP4 clusters showed a much broader size distribution ranging from 20 nm up to more than 160 nm (Fig. 4C). This observation strongly suggests that colocalizing CHMP4 protein assemblies form inside a restricting structure such as the budding neck whereas non-colocalizing CHMP4 clusters, which could not be correlated to nascent HIV-1 buds, have larger freedom in spatial spreading.

Some non-colocalizing CHMP4 protein assemblies formed diffuse clusters at the membrane that were significantly larger than the spatial resolution obtained under our conditions (circles, Fig. 4A). These large clusters were also observed in cells transfected with CHMP4B-HA in the absence of HIV-1 (S6B Fig.) but not in control experiments where the HeLa cells were not expressing CHMP4B-HA (S6A Fig.). These clusters were not considered in further analyses.

Again, we performed experiments in HeLa cells co-expressing the HIV:HIV^mCherry^ late- mutant (1:1) together with CHMP4B-HA (S6C Fig.). We observed a reduction in the total number of CHMP4B clusters as well as in the number of clusters colocalizing with the HIV^mCherry^ late- mutant (only 1 colocalization was observed for over 200 HIV-1 late- assembly sites). This is consistent with what is expected for a partially recruitment defective Gag variant containing a disrupted PTAP late- motif, supporting that CHMP4B-HA behaves similar to the untagged protein under our conditions. Analysis of the size distribution of the non-colocalizing CHMP4B structures in the late- experiments showed a similar distribution to non-colocalizing structures in experiments with wildtype HIV-1, which is broader than observed for the structures that colocalized with wildtype HIV-1 (Fig. 4B-4D).

### Super-resolution imaging of CHMP2A lattices colocalizing with HIV-1 assembly sites

**Fig 5:**
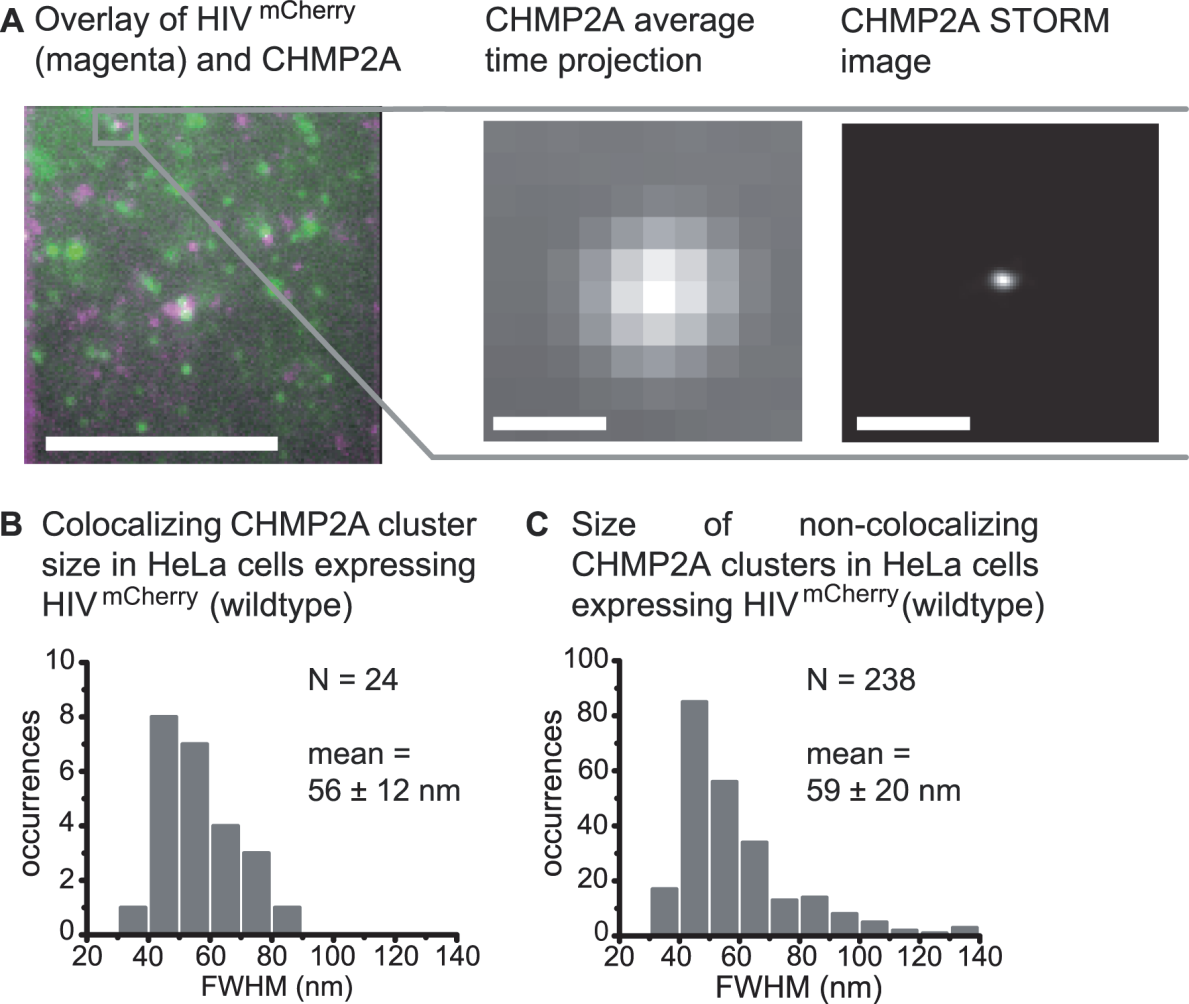
Super-resolution imaging of CHMP2A at HIV-1 assembly sites. HeLa cells were transfected with HIV:HIV^mCherry^ and endogenous CHMP2A was detected by immunostaining 14–15 h post transfection. HeLa cells were imaged using TIRFM. (**A**) *Left panel*. A merged time-projected TIRF image of HIV^mCherry^ (magenta) and CHMP2A (green) prior to STORM analysis. *Middle panel*. A zoomed-in image of the selected CHMP2A cluster highlighted in grey in the left panel colocalizing with an HIV assembly site. *Right panel*. The corresponding drift-corrected STORM image of the CHMP2A cluster. Scale bars: 500 nm. (**B**) The size distribution of all CHMP2A structures colocalizing with HIV^mCherry^ with an average cluster size (FWHM) of 56 ± 12 nm. (**C**) Size distribution of all non-colocalizing CHMP2A clusters in cells co-expressing HIV^mCherry^ with an average cluster size (FWHM) of 59 ± 20 nm.

We also performed super-resolution microscopy with CHMP2A, which is the last ESCRT protein recruited to the HIV-1 budding site and, in turn, recruits VPS4. Endogenous CHMP2A proteins were directly visualized using primary polyclonal anti-CHMP2A antibodies and secondary anti-bodies fluorescently labeled for dSTORM imaging. TIRF images of fixed cells revealed, similar to the previous experiments, distinct single HIV-1 budding sites and compact CHMP2A protein assemblies at the membrane that either colocalized with the HIV-1 assembly sites or not (Fig. 5A). The colocalization of CHMP2A with respect to HIV-1 assembly sites was 2.0%, similar to the values determined for Tsg101 and CHMP4B but smaller than for ALIX.

Super-resolution STORM images of CHMP2A clusters at HIV-1 assembly sizes revealed small, condensed spots (Fig. 5A) that showed a size distribution (FWHM) smaller than what we observed for Tsg101, the central spot of ALIX clusters and CHMP4B. It ranged from 38 to 82 nm with a mean value of 56 ± 12 nm (Fig. 5B). This value is in good agreement with the size of CHMP2 structures found in *in vitro* EM measurements by Effantin *et al*. [72]. In control experiments using untransfected HeLa cells, STORM imaging of the CHMP2A clusters showed condensed, circular structures at the cell membrane (S7A Fig.). Analysis of the size distribution of the non-colocalizing CHMP2A clusters reveals a broader size distribution than determined from the colocalizing structures ranging from 36 nm up to 138 nm (Fig. 5C) with a mean size of 59 ± 20 nm. Again, a narrower distribution of colocalizing CHMP2A clusters suggests that the cluster size is limited by the HIV-1 budding neck.

### STORM imaging of FP-tagged Tsg101 clusters at HIV-1 assembly sites

The cluster sizes determined here for all cellular proteins investigated differ significantly from those reported by Van Engelenburg *et al*. [47] and Bleck *et al*. [73]. In order to define the reason behind the apparent discrepancy, we performed control experiments employing FP-tagged variants of Tsg-101 and either labeled Gag in the full viral context (HIV:HIV^mCherry^) or labeled Gag alone (Gag:Gag.mCherry). Similar to what we observed for endogenous Tsg101, microscopy of cells co-transfected with YFP-Tsg101 fusions in the context of the full viral construct HIV:HIV^mCherry^ (1:1) [48] revealed condensed circular structures (S8A–S8B Fig.). Most strikingly, we detected a much higher colocalization rate between HIV^mCherry^ and YFP-Tsg101, detected using anti-Tsg101 antibodies, increasing from 1.8% up to ~18%. This effect was even stronger when HIV:HIV^mCherry^ was replaced by Gag:Gag.mCherry alone. The number of colocalizations between Gag.mCherry and immunostained YFP-Tsg101 (S8D–S8E Fig.) increased by over a factor of 10 up to 30% compared to results obtained for endogenous Tsg101. Though the increase in colocalization is mostly like induced by changes in the dynamics of the ESCRT interaction, it is conceivable that the higher fraction of colocalizations is due to better accessibility of the antibody epitope in the FP-Tsg101 clusters. In either case, there is a clear impact of the FP labeling. The dSTORM images of the YFP-Tsg101 clusters when coexpressed with either HIV:HIV^mCherry^ or Gag:Gag.mCherry showed condensed circular structures of 60 ± 19 nm and 60 ± 10 nm, respectively (S8C and S8F Fig.). The value for YFP-Tsp101 in the presence of Gag:Gag.mCherry is similar to what we observed for endogenous Tsg101 clusters in the context of full viral construct HIV^mCherry^ (Fig. 2C). To ensure that the increase in colocalization is not an artifact due to over expression of Tsg101, we also transiently expressed Tsg101-FLAG-IRES-GFP in HeLa cells. The small FLAG tag has been shown not to influence HIV-1 budding [74] and the IRES-GFP tag allowed us to identify the cells that were overexpressing Tsg101. The percentage of HIV-1 assembly sites with a colocalizing Tsg101 cluster (S4D Fig.) was similar to the value for endogenous Tsg101 (2.9%) and there was no detected difference in the size of the Tsg101 clusters (S4E–S4F Fig.).

### Two-color super-resolution imaging of HIV^mEos^ and CHMP4B-HA

For confirmation, we directly compared the size of the Gag shell of the HIV assembly site and the CHMP4B structure in the same bud by performing a combined STORM/PALM experiment in cells expressing CHMP4B-HA and HIV:HIV^mEos^. In the super-resolution image reconstruction of the CHMP4B and HIV^mEos^ TIRF images (S9A Fig.), the spot-like CHMP4B cluster in this case was situated on the rim of the HIV bud. The overlay of both super-resolution images (S9B Fig.) clearly illustrates the factor of two size difference between the CHMP4B protein assemblies and the HIV-1 bud as supported by the differences in the corresponding FWHM of the Gaussian fits (S9C Fig.).

### Comparison of our results with different ESCRT models

We have used super-resolution imaging of immunostained endogenous ESCRT components to investigate the architecture of ESCRT complexes involved in HIV budding and release. The super-resolution images of Tsg101, ALIX punctae, CHMP4B and CHMP2A membrane clusters that colocalized with HIV-1 budding sites all revealed a similar size distribution with an average cluster diameter that was significantly smaller than the average diameter of an HIV-1 bud. This is consistent with the observation that ALIX can function as a scaffold for stabilizing CHMP4 filaments within the neck as previously reported from *in vitro* experiments [30]. The most striking difference between our experiments with endogenous ESCRT proteins or CHMP4B-HA and other measurements using fluorescent protein fusions of the ESCRT machinery is the very low fraction of HIV-1 assembly sites with colocalizing ESCRT complexes. The transient interaction of ESCRT proteins on the timescale of a few minutes [13,60] with nascent assembly sites is expected to lead to the low percentage of colocalizations.

Using deep-etch electron microscopy of endogenous ESCRT proteins in VPS4 depleted cells, Cashikar *et al*. [40] observed flat rings or conical spirals with an average diameter of 108 ± 30 nm surrounding either a central membrane protrusion of ~ 50 nm diameter or Gag assembly sites. In our studies, we did not observe any ESCRT I (Tsg101), ALIX or ESCRT-III (CHMP4B and CHMP2A) protein structures colocalizing with HIV-1 assemblies sites with average sizes of 100 nm or larger. The larger cluster size may be attributable to the depletion of VPS4 used by Cashikar *et al*., which was necessary to increase the number of colocalizations between ESCRT factors and HIV assembly sites for electron microscopy. To check this possibility, we performed dSTORM experiments with CHMP2A in the presence of dominant negative VPS4 mutant. As expected, the number of colocalizations increased from 2.0% to 8.5%. In addition, we observed occasional colocalizing structures with sizes above 100 nm (S7C–S7E Fig.) similar to what we observed for non-colocalizing protein clusters at the membrane. The non-colocalizing structures are most likely linked to other cellular processes such as exosome activity. Therefore, the confined size distribution (FWHM) of less than ~60 nm for the various ESCRT protein clusters we measured is most likely determined by the constriction into scaffold structures smaller than the bud, such as the budding neck. Due to the low colocalization percentages in our experiments, the statistics are low and we cannot conclude with certainty that each ESCRT component is restricted to the neck region of the HIV-1 assembly site. However, the fact that no larger complexes were observed for all the ESCRT proteins investigated is a much stronger observation and supports an internal constriction mechanism where most of the ESCRT components are situated close to or within the HIV-1 budding neck (Fig. 1B, Model 2) [7,38] rather than protein structures restricting the bud neck from outside (Model 1).

A recent study using iPALM reported ESCRT protein clusters with diameters > 100 nm inside the Gag shell [47] which points towards a membrane constriction coming from within the bud (Model 3). As the structures we measured are significantly smaller than those from Van Engelenburg *et al*., the difference cannot be attributed to our two-dimensional measurements or differences in lateral resolution of 38 nm for our measurements versus their calculated iPALM resolution of 25 nm. The difference cannot be attributed to insufficient labeling as we have demonstrated our ability to label structures inside the bud using eGFP.vpr and antibodies against GFP (S3 Fig.). The major differences between our experiments and the results from Van Engelenburg *et al*. is their use of exogenous expressed FP-tagged versions of the ESCRT proteins, while we have, in most cases, performed immunostaining of endogenous proteins in the context of the full virus genome. It has been shown that ESCRT factors are very sensitive to the incorporation of additional tags that can affect the structure [33,40] and functionality [11] of the complexes. The results from our control experiments using YFP-tagged Tsg101 demonstrated an over 10-fold increase in the number of ESCRT complexes colocalizing with the Gag assembly sites (S1 Table). However, we observed no significant difference in cluster sizes for the YFP-Tsg101 construct with Gag.mCherry (60 ± 10 nm FWHM, 89 ± 26 nm for the Ripley’s analysis) compared with endogeneous Tsg101 colocalizing with HIV^mCherry^ (60 ± 19 nm FWHM, 85 ± 27 nm for the Ripley’s analysis). The increase in the colocalization of ESCRT factors with HIV-1 assembly sites when using fluorescent protein-tagged ESCRT conjugates demonstrates that there may be differences in recruitment dynamics and potentially structure even when it was reported that the constructs showed no detectable difference in phenotype. In addition, Van Engelenburg *et al*. detected significant amounts of eGFP-Tsg101, eGFP-CHMP2A and eGFP-CHMP4B proteins inside released viral particles by their eGFP fluorescence intensity and by immune-gold staining of cryo-TEM images using anti-eGFP antibodies. These results are inconsistent with the low amount of ESCRT proteins detected in released HIV-1 virus like particles as determined using western blot and mass spectrometry [11,17]. In recent experiments by Bleck et al [73] co-expressing Gag alone with FP-tagged ESCRT proteins, ESCRT proteins were observed to be significantly shifted with respect to the Gag assembly, inconsistent with ESCRT proteins being located in the lumen of the VLP (model 3). Hence, we also analyzed the position of the ESCRT complexes with respect to the center of the HIV-Gag signal. Consistent with the findings of Bleck et al [73], we often observed the ESCRT complexes on the edge, rather than the center, of the HIV bud (S10 Fig.). Hence, our data also support the model of ESCRT proteins being localized in the neck region of the HIV bud (model 2) rather than being incorporated into the bud itself (model 3).

As the cluster sizes we are measuring are not much larger than our localization precision of ~ 40 nm (S2 Table), the question arises regarding what impact our measurements could have on the actual sizes of the clusters. In super-resolution microscopy, as in normal optical microscopy, the final image is a convolution of the actual structure with the point-spread function of the optical system. For super-resolution microscopy, the point-spread function is determined by the localization precision. Therefore, the actual size of the ESCRT clusters will be smaller than the measured value. In addition, we have used primary and secondary full antibodies for labeling ESCRT proteins for STORM imaging. This can contribute up to an additional 30 nm (15 nm per antibody) to actual size of the ESCRT cluster [75]. Thus, the actual size of the ESCRT protein clusters at HIV-1 assembly sites is significantly smaller than our measured 60 nm, which would be closer to the late bud neck diameter and certainly lies below the diameter of ~50 nm for the central membrane protrusions observed by Cashiker *et al*. [40]. To distinguish between different models for ESCRT-III arrangement within the neck, 3D images with resolution of much better than ~ 40 nm will be necessary. As discussed above for the ESCRT machinery, the actual size of the HIV-1 clusters is also convoluted with the localization precision of the PALM experiments. Even though the PALM experiments had a lower resolution (~70 nm) than the STORM experiments, the correction factor for HIV-1 is similar or smaller than for the ESCRT complexes due to the larger size of the HIV-1 buds. In addition, the size of the FP tag is much smaller than the size added to the detected ESCRT complexes when using the full primary and secondary antibodies for labeling. A simple estimation of the corrected HIV-1 particle size yields a FWHM of ~ 95 nm rather than 116 nm, which is still significantly larger than the size of the observed ESCRT clusters.

In the case of ALIX, additional molecules were often (76%) detected in a cloud surrounding the central dense cluster. These cloud-like structures were exclusively found in colocalizations with HIV-1 assembly sites. HeLa cells not expressing HIV-1 exhibited only condensed spot-like clusters with a size and shape comparable to the central ALIX spots of colocalizing constructs in pCHIV-transfected cells. These results support the previous observation that ALIX is also located at the plasma membrane of uninfected cells [61,63]. A Ripley L-function cluster analysis of these cloud distributions revealed a clear correlation of the ALIX cloud to the size of a nascent virus particle, indicating that the diffuse, cloud-like ALIX distribution is based upon individual ALIX proteins being incorporated into budding Gag shells. This is consistent with previous studies that have shown that ALIX is present in released virus particles using a western blot assay [11,17]. Accumulation of ALIX at the budding site during virus assembly has been previously visualized in live-cell experiments with equine infectious anemia virus [13]. In contrast, transient recruitment of FP-tagged ALIX was at the end of HIV-1 bud assembly has recently been described [60]. Less than 20% of the recruited ALIX proteins were found remaining in the released viruses after fission [60], consistent with the relatively low number of ALIX proteins we detect in the cloud-like structures. Even though both Tsg101 and ALIX are known to bind to the p6-domain of Gag [17,19], we did not observe any cloud-like structures for Tsg101. Thus, the persistence of ALIX at the budding site even after fission may suggest that ALIX has an additional role beyond recruitment of ESCRT factors to the budding site of HIV-1.

### Conclusions

In summary, by using super-resolution fluorescence imaging of endogenous CHMP2 and HA-tagged CHMP4, we provide evidence for a membrane scission process driven from inside the HIV-1 budding neck by ESCRT-III protein assemblies including CHMP4B and CHMP2A. Protein recruitment factors acting early in the HIV-1 budding process such as Tsg101 and ALIX were also located in condensed clusters similar to the dimension of the neck and significantly smaller the HIV-1 bud. In addition, ALIX showed diffuse localizations surrounding the central cluster within the neck that did not extend the calculated dimension of the bud, indicating the internalization of individual ALIX proteins into the nascent HIV-1 particle. The low number of colocalizing events of recruited endogenous Tsg101, ALIX, CHMP2 and even of overexpressed HA-tagged CHMP4 with HIV-1 assembly sites supports the previously reported transient recruitment of those factors during virus particle membrane assembly rather than the accumulation of ESCRT proteins inside the bud. This stands in contrast to the much higher numbers of colocalization that was observed for the larger eGFP-Tsg101 protein fusion constructs and may lead to a higher accumulation probability of ESCRT proteins into the HIV-1 assembly sites. The resolution of STORM imaging is not yet high enough to resolve details below the diameter of the budding neck. Hence, the question is still open whether CHMP4 together with other ESCRT-III components form a spiral, whorl or a dome-like structure. The ongoing development of new labeling strategies and imaging techniques has the potential to answer this question and eventually unveil the membrane scission mechanism in the near future.

## Materials and Methods

### Plasmids

HIV encoding plasmid pCHIV and its labelled derivatives HIV^eGFP^, HIV^mCherry^ and HIV^mEos^ have been previously described [48,76]. The Vps4A-E228Q-mCherry plasmid was also described previously [45]. Plasmid synGag was obtained from Ralf Wagner (University of Regensburg, Germany)[77], pGag.eGFP was contributed by Marilyn Resh [59], and peGFP.Vpr was kindly provided by Tom Hope [78]. pGag.mCherry was derived from pGag.eGFP by replacing a BamHI/BsrGI fragment comprising the eGFP coding sequence with a corresponding restriction fragment comprising the mCherry ORF generated by PCR. The pCHMP4B-HA plasmid encoding CHMP4B fused to an HA-tag was kindly provided by H. Göttlinger [79]. The YFP-Tsg101 plasmid was a kind gift of Wesley Sundquist (University of Utah, Salt Lake City, USA).

### Cells and transfection

HeLa cells (Japanese Collection of Research Bioresources Cell Bank, Osaka Japan) were grown in Dulbecco’s modified Eagle’s medium (DMEM), supplemented with 10% fetal calf serum (FCS). HeLa cells were seeded into LabTek II 8-well chamber slides at a cell density of 2*10^4^ cells/well and transfected on the following day using X-tremeGENE transfection reagent (Roche) according to the manufacturer’s instructions. For the CHMP4B-HA/ HIV:HIV^mCherry^ experiments, 100 ng of CHMP4B-HA, 50 ng of pCHIV (wildtype) and 50 ng of pCHIV^mCherry^ were transfected per well. For the study of endogenous ESCRT proteins Tsg101, CHMP2A and ALIX, 50 ng pCHIV and 50 ng pCHIV^mCherry^ were used or, for PALM imaging of the HIV-1 assembly sites, 50ng pCHIV and 50 ng pCHIV^mEos^ were used. After transfection, cells were incubated for 14–15 hours at 37°C and 5.0% CO_2_.

For validating immunostaining efficiency within the viral bud, 75 ng of pEGFP.vpr and 50 ng of synGag (wildtype) and 50 ng of Gag.mCherry or Gag.eGFP were used for transfection, respectively. For experiments with Tsg101-fusion proteins, 50 ng of YFP-Tsg101 and 50 ng of pCHIV^mCherry^ and pCHIV were used for transfection, respectively. In the case of Gag.mCherry, the pCHIV plasmids were replaced by 50 ng synGag and 50 ng of Gag.mCherry, respectively.

For Vps4A-depletion experiments, 70 ng pCHIVeGFP, 70 ng pCHIV and 35 ng Vps4A-E228Q-mCherry were used for imaging of CHMP2A and cells were transfected with 50 ng CHMP4B-HA, 35 ng pCHIVeGFP, 35 ng pCHIV and 35 ng Vps4A-E228Q-mCherry for imaging CHMP4B-HA.

### Super-resolution sample preparation

For STORM imaging, immunostaining of Tsg101 was performed using primary mouse monoclonal anti-Tsg101 (clone 4A10, #GTX70255; Genetex) and secondary donkey anti-mouse IgG (#ABIN336468, purchased via antibodies-online.com) antibodies. For labeling of secondary anti-mouse antibodies, the unconjugated antibody was mixed with Alexa Fluor 488 succinimidyl ester (Invitrogen) and Cy5 bis-NHS-ester (GE Healthcare) in a molar ratio of 1:4:1 (antibody: Alexa Fluor 488: Cy5) in 150 mM NaHCO_3_ buffer (pH 8.2) and incubated overnight. Unreacted dye molecules were subsequently removed by gel permeabilization chromatography (Performa DTR Gel Filtration Cartridges from Edge BioSystems). For dSTORM experiments, secondary antibodies were only labeled with Cy5-bis-NHS-ester in a molar ratio dye:protein = 1:4.

Monoclonal primary mouse anti-ALIX antibodies (clone 3A9, #634502, BioLegend) were directly labeled for immunostaining of ALIX according to the protocol above as was also done for monoclonal primary anti-HA antibodies (clone 3F10, #11867423001; Roche) that were used to stain CHMP4B-HA.

For immunostaining of CHMP2A, we used unlabeled primary polyclonal rabbit anti-CHMP2A antibodies (#ab76335, abcam) combined with secondary donkey anti-rabbit IgG (#ABIN376979, purchased via antibodies-online.com) that were labeled according to the protocol above. Anti-GFP primary polyclonal antibodies from rabbit (#ABIN121945, purchased via antibodies-online.com) were used for labeling of peGFP.Vpr fusion protein together with the same secondary anti-rabbit IgG antibodies than were used for CHMP2A.

STORM sample preparation followed a slightly modified version of the protocol for microtubule immunostaining for STORM imaging described in [80]. HeLa cells were fixed not earlier than 14–15 h post transfection using 4% (v/v) paraformaldehyde solution (PFA, Electron Microscopy Sciences) for 15 min. Cells were permeabilized with 1% (v/v) Triton X-100 for 2 min. The cells were again rinsed twice with PBS. Unspecific antibody binding was blocked by incubating the sample with a buffer containing 3% bovine serum albumin (BSA) and 0.2% Triton X-100 for 30 min followed by immunostaining with primary antibodies for 60 min. In case of additional labeling with secondary antibodies, the sample was then stained with labeled secondary antibodies for 45 min.

Finally, post-fixation was done by treating the cells with 4% (v/v) paraformaldehyde solution in PBS for 10 min. STORM and dSTORM measurements were carried out using a glucose oxidase based oxygen scavenging buffer with mercaptoethylamine (Fluka) as reducing agent as described in [80]. PALM measurements were carried out using PBS buffer.

### Data acquisition

TIRF imaging and all PALM, STORM and dSTORM measurements were carried out on a combined TIRF and wide-field (WF) microscope as depicted in S1 Fig. A 561-nm diode-pumped solid-state laser (CrystaLaser, Reno, NV, USA) was used to excite Gag.mCherry and the red state of Gag.mEos. For photoconversion of mEosFP, we used a 405-nm diode laser (LuxX 405–120, Omicron Laserage, Rodgau, Germany). Excitation of GFP- and YFP-fusion proteins and activation of Cy5 for STORM imaging was achieved by exciting Alexa488 with a 488-nm diode laser (LuxX 488–60, Omicron Laserage, Rodgau, Germany). Activated Cy5 was excited by a 642-nm diode laser (PhoxX 642, Omicron Laserage, Rodgau, Germany). We performed wavelength selection by coupling the different laser lines into an acousto-optic tunable filter (TF525-250-6-3-GH18A, Gooch & Housego, Ilminster, UK). A set of two lenses then expanded the excitation beam in order to increase the field-of-view illuminated by TIRF excitation, before the light was focused on the back focal plane of a 100x N.A. 1.49 Apo TIRF oil immersion lens (Nikon, Tokyo, Japan). A rectangular glass prism introduced into the beam path after the focusing lens allowed switching between wide-field to TIRF imaging by changing the displacement of the beam path relative to the optical axis of the objective. A dichroic mirror (Di01-R405/488/561/635-25x36, Semrock, Rochester, NY, USA) then directed the excitation light into the microscope body, which consisted of a home-built microscope stage built out of Inwar to reduce thermal drift.

After passing the dichroic mirror, the collected fluorescence signal passed a set of emission filters to select a specific fluorescence wavelength: a 670/30 bandpass filter was used for Cy5 (Laser 2000), a 535/22 bandpass filter for GFP and YFP (FF01-535/22-25, Semrock, Rochester, NY, USA) and a 593/40 bandpass filter (FF01-593/40-25, Semrock, Rochester, NY, USA) for the red state of Gag.mEos and Gag.mCherry. Finally, the fluorescence signal was projected onto an EMCCD camera (DU860D-CS0-BV, Andor, Belfast, UK). The resulting pixel size was 120 nm.

ESCRT proteins were measured with either STORM or dSTORM, where a movie stack comprising 10,000 frames was acquired at a frame rate of 20 Hz, where each activation cycle consisted of one activation frame (λ_exc_ = 488 nm), followed by 9 imaging frames (λ_exc_ = 642 nm). Laser power at the output of the objective was 50 mW for imaging. For activation, the power of the blue laser was ~0.5 μW at the beginning of data acquisition for STORM and 5.0 μW for dSTORM and successively increased over the course of the experiment up to ~ 2 mW. This was necessary to counteract gradual bleaching of the activator dye. For PALM measurements of viral Gag protein, an analogue protocol was used with λ_exc_ = 405 nm for activation (increasing power starting at 5.0 μW to 2 mW) and λ_exc_ = 561 nm (1.1 mW) for imaging.

### Data analysis

The data analysis protocol is based on the procedure described by Rust *et al*. [81] and by Bates *et al*. [82] and implemented into a self-programmed analysis software using MATLAB (MathWorks, Natick, MA, USA). The same algorithms were used for PALM, STORM and dSTORM experiments. First, structures in the fluorescent image representing local maxima were isolated in a square 11 x 11 pixel window. The point spread function of the fluorescent molecule was evaluated by fitting these regions to a continuous ellipsoidal 2D Gaussian using Levenberg-Marquard’s nonlinear least-squares algorithm. The intensity *I* of the Gaussian distribution at coordinates (x, y) is given by:

$$I\left(x,y\right)\;=\;A+\;I_{0}e^{0.5*\;\left(-{\left(\frac{(x-x_{0}}{\sigma_{x}}\right)}^{2}-{\left(\frac{(y-y_{0}}{\sigma_{y}}\right)}^{2}\right)}$$

where *A* is the background intensity, $I_{0}$ the maximum amplitude of the distribution, $x_{0}$ and $y_{0}$ the coordinates of the centroid and $\sigma_{x}$ and $\sigma_{y}$ the standard deviations in x and y-direction, respectively.

Sample drift was corrected by pixel-wise cross-correlation of each frame *n* of the image stack with the first frame of this stack. The normalized cross-correlation function $G_{n}\left(x,\;y\right)$ for each frame is given by:

$$G_{n}\left(x,\;y\right)\;=\;\frac{\sum_{i}\sum_{j}\left(I_{1}\left(i,j\right)\right)\left(I_{n}\left(i+x,j+y\right)\right)}{{\left[\sum_{i}\sum_{j}{\left(I_{1}\left(i,j\right)\right)}^{2}\sum_{i}\sum_{j}{\left(I_{n}\left(i+x,j+y\right)\right)}^{2}\right]}^{0.5}}$$

The intensity $I_{1}$ from the first frame at coordinates (*i*, *j*) is correlated with the intensity $I_{n}$ from the *n* ^th^ frame at coordinates (i+x, j+y). The respective drift in *x*- and *y* is given by the position in (x, y) of the maximum of $G_{n}\left(x,\;y\right)$. In order to reduce fluctuations caused by fluorophore blinking, the obtained drift function was fitted to a polynomial and the fit function used for drift correction.

We rejected all molecules where the Gaussian function fitted to the point spread function showed an ellipticity, *E*, higher than 15% (S11A Fig.). *E* depends on the Gaussian standard deviations in *x* and *y*-direction (σ_x_ and σ_y_) and is defined as:

$$E=\left|\frac{\sigma_{x}-\sigma_{y}}{\sigma_{x}+\sigma_{y}}\right|$$

Points that appeared in two or more consecutive frames within a distance smaller than 1 px (120 nm) were considered as originating from the same fluorescent molecule. The positions determined from individual frames where the same molecule was observed were averaged for rendering of the final super-resolution image. Diffraction limited spots that appeared for only one frame or molecules with less than 300 detected photons were discarded.

Localization displacements of single fluorophores molecules were used to estimate the actual resolution of our system by means of the FWHM value of the Gaussian function fit to the resulting displacement histograms in the *x*- and *y*-direction respectively (S11B Fig.). The average STORM image resolutions for the different analyzed proteins are summarized in S2 Table.

For the final STORM image rendering with a pixel size of 12 nm in the super-resolution images, each localized molecule was represented by a 2D Gaussian function with a fixed amplitude I_0_ = 1000 counts and a fixed standard deviation σ_x_ = σ_y_ = 1.2 px = 14.4 nm.

Diffraction limited TIRF images of (d)STORM/PALM measurements, which were used to identify colocalizations of ESCRT and HIV-1 buds, were emulated by building the average time projection of the acquired image stack.

An self-written image analysis algorithm was developed in ImageJ Macro language [83] and used to consistently assess the number of HIV-1 assembly sites and clusters and determine their colocalization. Images were individually analyzed as follows: First, a convolution filter (Gaussian blur) followed by background subtraction (“rolling ball” algorithm [84]) were applied. Next, point objects were selected based on their intensities (local maxima) and a multi-point selection was created. Objects corresponding to either assembly sites or clusters were then segmented by a watershed approach. As a last step, center of brightness, distribution of intensities and area of each object were measured. These readouts were collected, analyzed accordingly and generated the statistical information presented in Figs. 2–5. Gag assemblies with an area > 0.860 μm² were excluded from further evaluation.

ESCRT clusters were counted by rendering a STORM image with a pixel size equal to the widefield pixel size of 120 nm and all clusters with an intensity lower than 5,000 counts were discarded as well as all clusters where no distinct structure could be obtained in the final STORM image with a pixel size of 12 nm.

Object sizes in the final STORM or PALM images were either estimated by means of the average full-width at half-maximum (FWHM) of 1-D Gaussians fitted to two orthogonal 1-D cross-sections through the middle of the respective cluster as demonstrated in S2B, S4B, S5B, S6D and S7B Figs., or alternatively using Ripley’s L-test [49]. In the case of estimation of the size of the cloud surrounding ALIX clusters, the points contributing to the central clusters were excluded from Ripley’s analysis to achieve more accurate results for the size of the cloud (see S5C Fig.).

## Supporting Information

S1 File Supplementary results. Size of HIV-1 budding sites using Photoactivation Localization Microscopy (PALM)
(DOCX)

S1 Fig Schematic of the custom microscopy setup used for super resolution imaging (green: excitation pathway; yellow: detection pathway). (EPS)

S2 Fig Super-resolution imaging of HIV-1 assembly sites. Cells were transfected with equimolar amounts of HIV^mEos^ and untagged HIV. (**A**) *Left panel*. Time projected TIRF image of HIV^mEos^ showing various HIV-1 assembly sites. Scale bar: 10 μm. *Middle panel*. A zoomed-in TIRF image of a single HIV-1 assembly site highlighted by the red box in panel **A**. Scale bar: 500 nm. *Right panel*. Drift-corrected super-resolution PALM image of the HIV-1 assembly site shown in panel **B**. Scale bar: 500 nm. (**B**) Gaussian fits of the cross-sections (red lines) through the PALM reconstructed point cluster shown in the right panel of **A**. (**C**) The size distribution of *N* = 159 HIV^mEos^ clusters determined from the FWHM of the Gaussian fit of the super-resolution images is shown with an average size (FHWM) of 116 ± 36 nm.
(EPS)

S3 Fig eGFP.vpr measurements. In all images, eGFP.vpr expressed in HeLa cells was detected by immunostaining 18 – 20 h post transfection or directly via the eGFP fluorescence by TIRFM. (**A**) TIRFM images of a HeLa cell expressing both pEGFP.vpr and Gag.mCherry, and immunostained using antibodies against GFP, visualized in the eGFP channel (left panel), mCherry channel (middle panel) and anti-GFP (right panel) channel. Overlays of images demonstrating the colocalizations of eGFP.Vpr and HIV^mCherry^ (B) and colocalizations of eGFP.vpr and anti-GFP (**C**) (magenta: Gag.mCherry, yellow: eGFP.vpr, green: anti-GFP) are shown, scale bars: 10 μm. (**D**) Size distribution of eGFP.vpr clusters colocalizing with Gag.mCherry determined from the full-width at half-maximum (FWHM) obtained by fitting a Gaussian function to the cross-section through the respective eGFP.Vpr cluster. The average cluster size (FHWM) is 56 ± 12 nm. *N* represents the number of analyzed colocalizing clusters.
(EPS)

S4 Fig Tsg101 supplementary information. (**A**) An untransfected HeLa cell immunostained with anti-Tsg101 primary plus labeled secondary antibody binding to Tsg101 primary antibodies, scale bar: 10 μm. (**B**) The size of Tsg101 clusters was determined from the average full-width at half-maximum (FWHM) obtained by fitting cross sections of the cluster to two orthogonal 1-D Gaussian functions (indicated by the red lines). Scale bar: 500 nm. (**C**) TIRF images of Tsg101 in HIV:HIV^mCHerry^ late- expressing cells. *Left panel*. TIRF image of HIV^mCherry^ late- assembly sites. *Middle Panel*. Average time projection of TIRF image series acquired for super-resolution imaging of Tsg101 in HIV:HIV^mCherry^ late- expressing cells. *Right panel*: Overlay of the left (HIV^mCherry^ late-, magenta) and middle (anti-Tsg101, green) panels. Scale bars: 10 μm. No colocalizations are observed. (**D**) *Left panel*. A merged time-projected TIRF image of immunostained Tsg101-FLAG (green) in a HeLa cell expressing Tsg101-FLAG-IRES-GFP together with HIV^mCherry^ (magenta) and HIV prior to STORM analysis. *Middle panel*. A zoomed-in image of the selected Tsg101-FLAG cluster highlighted in grey in the left panel colocalizing with an HIV assembly site. *Right panel*. The corresponding drift-corrected STORM image of the Tsg101-FLAG cluster. Scale bars: 500 nm. (**E**) The size distribution of all Tsg101-FLAG structures colocalizing with HIV^mCherry^ with an average cluster size (FWHM) of 50 ± 11 nm. (**F**) Size distribution of all non-colocalizing Tsg101-FLAG clusters in cells co-expressing HIV^mCherry^ (wildtype) with an average cluster size (FWHM) of 51 ± 10 nm. *N* represents the number of analyzed colocalizing clusters.
(EPS)

S5 Fig ALIX supplementary information. (**A**) An untransfected HeLa cell immunostained with anti-labeled ALIX primary antibodies. All zoomed-in insets show drift-corrected super-resolution STORM images of the respective clusters highlighted in red. Scale bars: 10 μm (large image), 200 nm (insets). (**B**) Size characterization of the central, condensed spot of ALIX colocalizing with HIV^mCherry^; two orthogonal cross-sections through the center of the spot are fitted to a 1D Gaussian function whose mean FWHM represents the structure size. Scale bar: 500 nm. (**C**) Size characterization of the diffuse cloud-like ALIX structure from the example shown in panel **B**; the central cluster is masked (inset) and the cloud analyzed by applying Ripley’s L-function, whose maximum at 187 nm indicates the spreading size of the cloud cluster. Scale bar: 500 nm.
(EPS)

S6 Fig CHMP4B-HA control experiments. HeLa cells transfected with (**A**) only HIV:HIV^mCherry^ or (**B**) only CHMP4B-HA and immunostained with anti-HA primary antibodies. TIRF images of the HIV^mCherry^ channel and the CHMP4B-HA channel are shown in the *left* and *right* panels, respectively. All scale bars: 20 μm. (**C**) A HeLa cell transfected with HIV:HIV^mCherry^ late- and CHMP4B-HA. *Left panel*. A TIRFM image of HIV^mCherry^ late- assembly sites. *Middle panel*. A time-projected TIRFM image of the CHMP4B-HA channel before the STORM localization analysis. *Right panel*. An overlay of the two channels with HIV^mCherry^ channel shown in magenta and the CHMP4B-HA channel in green. No colocalizing structures are observed in the merged image. Scale bars: 10 μm. (**D**) *Left panel*. A STORM image of a CHMP4B cluster colocalizing with wildtype HIV^mCherry^. Scale bar: 500 nm. The size of CHMP4B-HA clusters was determined using the FWHM obtained by fitting 1D Gaussian functions to the orthogonal cross-sections. *Right panel*. The cross-sections, indicated by the red lines in the left panel, and the Gaussian fits are shown for comparison.
(EPS)

S7 Fig CHMP2A control experiments and analysis. (**A**) An untransfected HeLa cell immunostained with anti-CHMP2A primary and appropriate labeled secondary antibodies. All zoomed in images show drift-corrected super-resolution STORM images of the respective clusters highlighted in red. Scale bars: 10 μm (large image), 200 nm (insets). (**B**) A STORM image of a CHMP2A cluster. Scale bar: 500 nm. The size of CHMP2A clusters was determined from the mean of the full-width at half-maximum (FWHM) obtained by fitting a 1D Gaussian function to each of the cross-sections indicated by the red lines. (**C**) *Left panel*. A merged time-projected TIRF image of immunostained CHMP2A (green) in a HeLa expressing dominant-negative Vps4A mutant Vps4A-E228Q-mCherry together with HIV^mCherry^ (magenta) and HIV prior to STORM analysis. *Middle panel*. A zoomed-in image of the selected CHMP2A cluster highlighted in grey in the left panel colocalizing with an HIV assembly site. *Right panel*. The corresponding drift-corrected STORM image of the CHMP2A cluster. Scale bars: 500 nm. (**D**) The size distribution of all CHMP2A structures in cells expressing the dominant-negative Vps4A-E228Q-mCherry mutant colocalizing with HIV^mCherry^. The average cluster size (FWHM) is 66 ± 23 nm. (**E**) Size distribution of all non-colocalizing CHMP2A clusters in cells co-expressing HIV^mCherry^ (wildtype) and Vps4A-E228Q-mCherry with an average cluster size (FWHM) of 65 ± 22 nm. *N* represents the number of analyzed colocalizing clusters.
(EPS)

S8 Fig Experiments with YFP-tagged Tsg101. (**A**) A HeLa cell expressing the ESCRT fusion protein YFP-Tsg101 and HIV:HIV^mCherry^. Signal from the YFP channel (*left panel*), HIV:HIV^mCherry^ channel (*middle panel*) a time-projection of the immunostained Tsg101 signal labeled with primary anti-Tsg101 antibodies and fluorescently labeled secondary antibodies (*right panel*) are shown. Scale bars: 10 μm. (**B**) *Left panel*. A merged TIRFM image of HIV^mCherry^ (magenta) and immunostained YFP-Tsg101 (green). Scale bar: 10 μm. A high number of colocalizations are observed in the field of view. *Right panel*. A STORM image of the condensed, circular Tsg101 structure highlighted in the middle panel in grey. Scale bar: 500 nm. (**C**) The size distribution of immunostained YFP-Tsg101 clusters colocalizing with HIV^mCherry^ with an average size (FWHM) of 60 ± 19 nm. (**D**) A HeLa cell expressing the ESCRT fusion protein YFP-Tsg101 and Gag:Gag.mCherry. Signal from the YFP channel (*left panel*), Gag:Gag.mCherry channel (*middle panel*) a time-projection of the immunostained Tsg101 signal labeled with primary anti-Tsg101 antibodies and fluorescently labeled secondary antibodies (*right panel*) are shown. Scale bars: 10 μm. (**E**) *Left panel*. A merged TIRFM images of Gag.mCherry (magenta) and immunostained YFP-Tsg101 (green). A high number of colocalizations are observable in the field of view. Scale bar: 10 μm. *Right panel*. A STORM image of the condensed, circular Tsg101 structure highlighted in the middle panel in grey. Scale bar: 500 nm. (**F**) The size distribution of immunostained YFP-Tsg101 clusters colocalizing with Gag.mCherry with an average size (FWHM) of 60 ± 10 nm. *N* represents the number of analyzed colocalizing clusters.
(EPS)

S9 Fig Dual-color super-resolution imaging of CHMP4B and HIV-Gag. (**A**) Overlay of the average time projections of independent TIRFM image series for HIV^mEos^ and CHMP4B-HA (magenta: HIV^mEos^, green: CHMP4B-HA), scale bar: 2 μm. (**B**) A zoomed-in image of the single colocalizing structure (*upper panels*) and corresponding super-resolution PALM (mEOS) or STORM (CHMP4B-HA) images, respectively (*lower panels*), scale bars: 500 nm. (**C**) The Gaussian fit of cross-section profiles through the respective PALM/STORM images yielding a FWHM of 133 nm for HIV^mEos^ (*left graph*) and 57 nm for CHMP4B-HA cluster (*right graph*).
(EPS)

S10 Fig Position of ESCRT protein clusters relative to the HIV-1 bud. Overlay of super-resolution images of ALIX and ESCRT proteins Tsg101, CHMP4B-HA and CHMP2A (green) and the corresponding TIRF images of the respective evolving HIV-1 buds (magenta) reveal the relative position of the ESCRT protein cluster relative to the viral bud.
(EPS)

S11 Fig Ellipticity threshold and determination of image resolution. (**A**) Ellipticity distribution of fitted point spread functions and applied ellipticity threshold of 0.15. (**B**) Determination of the resolution of the system by displacement analysis of all summarized single localizations in the *x*-direction. The resolution is given by the FWHM of the localization displacement distribution (here: 38.3 nm). See S2 Table for the localization precision of all experiments.
(EPS)

S1 Table Summary of results for analyzed ESCRT-proteins Tsg101, ALIX, CHMP4B and CHMP2A regarding number of analyzed cells, cluster and colocalization events. (PDF)

S2 Table STORM image resolution for different analyzed proteins. (PDF)
