# Supplementary material for: Super-Resolution Imaging of ESCRT-Proteins at HIV-1 Assembly Sites
Source: PLoS Pathog. 2015 Feb 24;11(2):e1004677. doi: 10.1371/journal.ppat.1004677 (PMC4339578; doi:10.1371/journal.ppat.1004677)
Supplement: S1 File — Size of HIV-1 budding sites using Photoactivation Localization Microscopy (PALM) (DOCX) [file ppat.1004677.s001.docx]

**Super-Resolution Imaging of ESCRT-proteins at HIV-1 Assembly Sites**

**Jens Prescher, Viola Baumgärtel, Sergey Ivanchenko, Adriano A. Torrano, Christoph Bräuchle, Barbara Müller, Don C. Lamb**

**Supplementary Results**

**Size of HIV-1 budding sites using Photoactivation Localization Microscopy (PALM)**

To determine the essential difference between the two main membrane fission models [[1](#_ENREF_1),[2](#_ENREF_2)], the super-resolution images of ALIX, CHMP4B, CHMP2A and Tsg101 protein clusters need to be placed in the context of the actual dimensions of individual HIV-1 budding sites (S2A Fig.). Hence, even though the diameter of HIV-1 budding sites is known to be on the order of 130 nm from Cryo-EM studies on MT-4 cells [[3](#_ENREF_3),[4](#_ENREF_4)], we measured the diameter of individual budding sites using the experimental settings we applied for super-resolution imaging on our home-built TIRF/WF fluorescence microscope (S1 Fig.)**.** For this purpose, we used the previously described non-infectious full viral construct HIV^mEos^ [[5](#_ENREF_5)] containing the photoconvertible fluorescent protein mEos [[6](#_ENREF_6)] inserted between the matrix (MA) and capsid (CA) domains of Gag [[7](#_ENREF_7),[8](#_ENREF_8)] in a 1:1 mixture with the non-labeled, non-infectious viral construct HIV-1. PALM imaging was performed using TIRF excitation to reduce cellular background caused by autofluorescence. As expected, cells expressing HIV^mEos^ showed fluorescent punctae at the plasma membrane indicating newly formed HIV-1 budding sites (S2A Fig.). Super-resolution PALM images were constructed from the precise localization of many single Gag.mEos molecules at the HIV-1 buddings sites and revealed round, dense clusters of Gag proteins, as expected for the compact Gag shell formed during the assembly process (S2A Fig.**,** right panel). The full-width at half-maximum (FWHM) of the Gaussian function fitted to the cross-section of 159 HIV^mEos^ clusters was used to estimate the sizes of the underlying HIV-1 budding sites (S2B and S2C Fig.). In our experiments, the size distribution of an individual budding site ranged from 80 to 180 nm with a mean value of 116 ± 36 nm. A Ripley L-function analysis yielded a diameter of 141 ± 41 nm for the HIV-1 particles. These values are similar with the previously obtained values from super-resolution fluorescence imaging by Lehmann *et al.* (94 ± 37 nm) [[9](#_ENREF_9)], Eckhardt *et al.* (~ 140 nm) [[10](#_ENREF_10)], Malkusch *et al*. (~ 120 nm) [[11](#_ENREF_11)] and Gunzenhäuser *et al*. (106 ± 24 nm) [[12](#_ENREF_12)].

As fixation and permeabilization can alter the properties of membranes and thereby influence the size of the clusters, we also investigated the size distribution of HIV-1 assembly complexes in live cells. HeLa cells were cotransfected with pCHIV:pCHIV^mEos^ and imaged using PALM 24 hpt. The size distribution of 54 HIV-1 assembly sites is shown in S2D Fig. The results are similar to that for fixed cells with an average FWHM of 108 ± 35 nm and diameter determined using a Ripley L-function analysis of 152 ± 37 nm.

**Supporting Information References**

1. Hurley JH, Hanson PI (2010) Membrane budding and scission by the ESCRT machinery: it’s all in the neck. Nature Reviews Molecular Cell Biology 11: 556-566.

2. Hanson PI, Roth R, Lin Y, Heuser JE (2008) Plasma membrane deformation by circular arrays of ESCRT-III protein filaments. JCB 180: 389-402.

3. Carlson L-A, Briggs JAG, Glass B, Riches JD, Simon MN, et al. (2008) Three-Dimensional Analysis of Budding Sites and Released Virus Suggests a Revised Model for HIV-1 Morphogenesis. Cell Host & Microbe 4: 592-599.

4. Carlson L-A, Marco Ad, Oberwinkler H, Habermann A, Briggs JAG, et al. (2010) Cryo Electron Tomography of Native HIV-1 Budding Sites. PLoS Pathogens 6: e1001173.

5. Lelek M, Nunzio FD, Henriques R, Charneau P, Arhel N, et al. (2012) Superresolution imaging of HIV in infected cells with FlAsH-PALM. PNAS 109: 8564–8569.

6. Wiedenmann J, Ivanchenko S, Oswald F, Schmitt F, Röcker C, et al. (2004) EosFP, a fluorescent marker protein with UV-inducible green-to-red fluorescence conversion. PNAS 101: 15905–15910.

7. Müller B, Daecke J, Fackler OT, Dittmar MT, Zentgraf H, et al. (2004) Construction and Characterization of a Fluorescently Labeled Infectious Human Immunodeficiency Virus Type 1 Derivative. Journal of Virology 78: 10803–10813.

8. Lampe M, Briggs JAG, Endress T, Glass B, Riegelsberger S, et al. (2007) Double-labelled HIV-1 particles for study of virus–cell interaction. Virology 360: 92–104.

9. Lehmann M, Rocha S, Mangeat B, Blanchet F, Uji-i H, et al. (2011) Quantitative Multicolor Super-Resolution Microscopy Reveals Tetherin HIV-1 Interaction. PLoS Pathogens 7: e1002456.

10. Eckhardt M, Anders M, Muranyi W, Heilemann M, Krijnse-Locker J, et al. (2011) A SNAP-Tagged Derivative of HIV-1 - A Versatile Tool to Study Virus-Cell Interactions. PLoS ONE 6: e22007.

11. Malkusch S, Muranyi W, Muller B, Krausslich HG, Heilemann M (2013) Single-molecule coordinate-based analysis of the morphology of HIV-1 assembly sites with near-molecular spatial resolution. Histochem Cell Biol 139: 173-179.

12. Gunzenhäuser J, Olivier N, Pengo T, Manley S (2012) Quantitative Super-Resolution Imaging Reveals Protein Stoichiometry and Nanoscale Morphology of Assembling HIV-Gag Virions. Nano Lett 12: 4705-4710.
