# Supplementary material for: Super-Resolution Imaging of ESCRT-Proteins at HIV-1 Assembly Sites
Source: PLoS Pathog. 2015 Feb 24;11(2):e1004677. doi: 10.1371/journal.ppat.1004677 (PMC4339578; doi:10.1371/journal.ppat.1004677)
Supplement: S1 Table — (PDF) [file ppat.1004677.s013.pdf]

S1 Table: Summary of results for analyzed ESCRT-proteins Tsg101, ALIX, CHMP4B and CHMP2A regarding number of analyzed cells, cluster and colocalization events.

	Tsg101 + $HIV^{mCherry}$	ALIX + $HIV^{mCherry}$	CHMP4B-HA + $HIV^{mCherry}$	CHMP2A + $HIV^{mCherry}$	YFP-Tsg101+ $HIV^{mCherry}$	YFP-Tsg101+ Gag.mCherry	Tsg101 + $HIV^{mCherry\ (late\ -)}$	CHMP4B-HA + $HIV^{mCherry\ (late\ -)}$
Number of experiments	3	4	11	4	1	1	2	2
Number of analyzed cells	14	10	23	17	2	4	6	5
Number of detected HIV-1 buds	1052	529	1662	1128	87	194	210	235
HIV-1 buds colocalizing with ESCRT	1.8 %	3.4 %	1.5 %	2.0 %	18.3 %	29.5 %	0 %	0.4 %
Avg. colocalization cluster size (nm) (FWHM)	58 ± 7.1	64 ± 18	56 ± 12	56 ± 12	60 ± 19	60 ± 10	-	47
Protein clusters total	221	115	306	262	70	146	83	36
Protein clusters colocalizing with HIV budding site	18	17	23	24	16	55	0	1
Protein clusters not colocalizing with HIV budding site	203	98	283	238	54	91	83	35
Protein clusters enclosed by cloud	0	13	0	0	0	0	0	0
Size of cloud (nm)	-	164 ± 31	-	-	-	-	-	-
