# Supplementary material for: Super-Resolution Imaging of ESCRT-Proteins at HIV-1 Assembly Sites
Source: PLoS Pathog. 2015 Feb 24;11(2):e1004677. doi: 10.1371/journal.ppat.1004677 (PMC4339578; doi:10.1371/journal.ppat.1004677)
Supplement: S2 Table — (PDF) [file ppat.1004677.s014.pdf]

S2 Table: STORM image resolution for different analyzed proteins

Protein	STORM image resolution[a]
Tsg101	48.1 ± 10.3 nm (x)/51.1 ± 11.9 nm (y)
ALIX	36.1 ± 13.4 nm (x)/37.8 ± 10.3 nm (y)
CHMP4B	40.8 ± 8.4 nm (x)/43.6 ± 9.6 nm (y)
CHMP2A	46.0 ± 14.2 nm (x)/46.9 ± 12.7 nm (y)
HIVmEos	71.8 ± 11.7 nm (x)/76.6 ± 16.8 nm (y)

[a]The given resolution is the FWHM of the localization displacement distribution function determined from molecules observed in more than one consecutive image (S11B Fig.).
